# Risk of neurodevelopmental disorders in children born from different ART treatments: a systematic review and meta-analysis

**DOI:** 10.1186/s11689-020-09347-w

**Published:** 2020-12-13

**Authors:** Tono Djuwantono, Jenifer Kiem Aviani, Wiryawan Permadi, Tri Hanggono Achmad, Danny Halim

**Affiliations:** 1 Department of Obstetrics and Gynecology, Faculty of Medicine, Universitas Padjadjaran/Dr. Hasan Sadikin General Hospital, Bandung, West Java Indonesia; 2 Bandung Fertility Center, Limijati Mother and Child Hospital, Bandung, West Java Indonesia; 3 Department of Basic Medical Science, Faculty of Medicine, Universitas Padjadjaran, Bandung, West Java Indonesia; 4 Research Center for Medical Genetics, Faculty of Medicine, Universitas Padjadjaran, Bandung, West Java Indonesia

**Keywords:** Neurodevelopmental disorders, Long-term outcome, Cerebral palsy, Intellectual disability, Autism spectrum disorder, Assisted reproductive treatments, In vitro fertilization, Intracytoplasmic sperm injection, Frozen embryo transfer

## Abstract

**Background:**

Various techniques in assisted reproductive technology (ART) have been developed as solutions for specific infertility problems. It is important to gain consensual conclusions on the actual risks of neurodevelopmental disorders among children who are born from ART. This study aimed to quantify the relative risks of cerebral palsy, intellectual disability, autism spectrum disorder (ASD), and behavioral problems in children from different ART methods by using systematic review and meta-analysis. Healthcare providers could use the results of this study to suggest the suitable ART technique and plan optimum postnatal care.

**Methods:**

Pubmed, Google Scholar, and Scopus databases were used to search for studies up to January 2020. Of the 181 screened full manuscripts, 17 studies (9.39%) fulfilled the selection criteria. Based on the Newcastle-Ottawa scale ratings, 7 studies were excluded, resulting in 10 studies that were eventually included in the meta-analyses. Mantel-Haenszel risk ratio model was used in the meta-analysis, and the results are described using forest plot with 95% confidence interval. Heterogeneity was assessed using the *I*^2^ value.

**Results:**

Pooled evaluation of 10 studies showed that the risk of cerebral palsy in children from assisted reproductive technology (ART) is higher than children from natural conceptions (risk ratio [RR] 1.82, [1.41, 2.34]; *P* = 0.00001). Risk of intellectual disability (RR 1.46, [1.03, 2.08]; *P* = 0.03) and ASD (RR 1.49 [1.05, 2.11]; *P* = 0.03) are higher in intracytoplasmic sperm injection (ICSI) children compared to conventional in vitro fertilization (IVF) children. The differences in the risk of neurodevelopmental disorders in children born after frozen and fresh embryo transfers are not significant. Analysis on potential cofounder effects, including multiple birth, preterm birth, and low birth body weight highlight possibilities of significant correlation to the risks of neurodevelopmental disorders.

**Conclusions:**

Pooled estimates suggest that children born after ART are at higher risk of acquiring cerebral palsy. ICSI treatment causes higher risk of intellectual disability and ASD. These findings suggest the importance of the availability of intensive care unit at the time of delivery and long-term developmental evaluation particularly in children from ICSI.

**Supplementary Information:**

The online version contains supplementary material available at 10.1186/s11689-020-09347-w.

## Introduction

The dreams of many infertile couples to have offspring(s) have been made possible by assisted reproductive technology (ART). Through its constant development, morbidity due to infertility has been massively decreased [1]. Every technique, such as intracytoplasmic sperm injection (ICSI) and frozen embryo transfer, is designed as solutions for specific problems. For example, in cases where two previous conventional in vitro fertilization (IVF) failed, or if the majority of sperms are immotile, ICSI is the technique that offers the highest possibility to acquire pregnancy(-ies) [2]. Meanwhile, when couples preferred to obtain pregnancy(-ies) at later stage(s), cryopreservation enables them to freeze embryos for implantation at later times [3].

Studies on prenatal and postnatal outcomes in ART, such as the risks of multiple pregnancies and congenital malformations, have already been comprehensively reviewed and meta-analyzed on previous publications [4–10]. Conversely, lesser number of studies have focused on the neurodevelopmental aspects of children from ART. The nature of certain neurodevelopmental disorders that might not be visible until later stage in children’s lives, difficulties in obtaining long-term follow-up, and the fact that many disorders can only be diagnosed and treated in a multidisciplinary fashion might be some of the reasons that caused limitation in the number of studies on neurodevelopmental outcomes in these children. Nonetheless, several studies have been published to date, and it is important to gain consensual conclusions on the actual risks of neurodevelopmental disorders among these children.

This study aims to assess the risks of neurodevelopmental abnormalities in children from ART treatments, in comparison to naturally conceived children through meta-analysis. As different ART treatments may translate into different pathophysiology, the neurodevelopmental outcomes in children from different ART techniques are also compared.

## Methods

### Literature search and identification

This systematic review and meta-analysis was conducted according to the Preferred Reporting Items for Systematic Reviews and Meta-analyses (PRISMA) [11] reporting guidelines. PubMed, Scopus, and Google scholars’ databases were used to collect publications up to January 16, 2020. The following search terms were applied: reproductive techniques, assisted OR assisted reproductive OR in vitro fertilization OR in vitro fertilization AND Child development OR adolescent development OR long term follow-up OR Developmental disabilities OR Developmental disability OR Language disorders OR Language disorder OR Neurodevelopment OR mental disorder OR mental disorders OR neuropsychiatry. Additional studies were identified through screening of the reference lists.

### Inclusion and exclusion criteria

Studies were included if they (1) compared any neurodevelopment aspects in ART children to children from natural conceptions, and (2) described the neurodevelopment in children from common ART techniques, including conventional IVF, ICSI, cryopreserved, and fresh embryo transfers, then compared them to either children in general population or children conceived by different treatment modalities. Studies were excluded if (1) information were insufficient, particularly related to the subjects and parameters in the study, (2) not including original data, such as reviews, systematic reviews, comments, or editorial letters, (3) not including control group (e.g., case reports), or could not ascertain the used fertility treatment, and (4) not written in English.

### Data collection and analysis

The title and abstract of every article were independently reviewed by 3 authors (TD, JKA, DH). If the abstract met the inclusion criteria, the full-text article was thoroughly reviewed. Reference lists on the identified publications were screened for previously unidentified but relevant studies. The following information were retrieved: author, country, publication year, number of patients, criteria of neurodevelopmental disorders, and the patients’ neurodevelopmental outcomes. The quality of methodology was assessed using the risk of bias criteria that were specified in Newcastle-Ottawa Scale (NOS) [12]. Based on the final ratings, high-quality studies (NOS score > 7) were included in the meta-analyses.

### Statistical analysis

Meta-analysis was performed using Mantel-Haenszel risk ratio (RR) with 95% confidence interval for dichotomous data. Meta-analysis was performed on subgroup analysis based on the types of the acquired neurodevelopmental disorders, including cerebral palsy, intellectual disability, autism spectrum disorder, and behavioral problems. To describe the overall risk of neurodevelopmental disorders, a meta-analysis of the overall data was also conducted. The RevMan version 5.3 software (Cochrane Collaboration) was used for these purposes. Inconsistency index (*I*^2^) test, which ranges from 0 to 100%, was calculated to evaluate heterogeneity across studies. Values above 50% or *p* value < 0.10 indicate a significant heterogeneity. Regression-based Egger’s test was used to evaluate small study effect and the risk of publication bias. *p* values less than 0.05 were considered as significant bias. Cochrane Risk of Bias Assessment tool (Cochrane Collaboration) was used to assess the risk of bias. Certainty of the evidence quality was evaluated by using the Guideline Development Tool by GRADEpro.

## Results

**Fig. 1:**
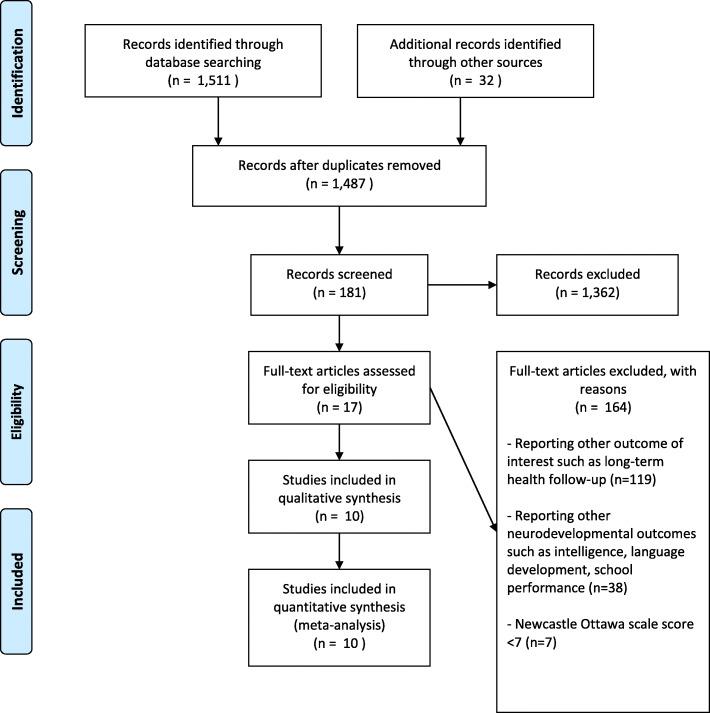
Study flow chart based on PRISMA guidelines

The literature searches identified 1511 studies. Additionally, 32 studies were identified through reference screening (Fig. 1). In total, 181 full-text articles were reviewed, and only 17 articles fulfilled the inclusion criteria. Based on the Newcastle-Ottawa scale ratings, 7 studies [13–19] were excluded. Thus, 10 studies [20–29] were included in the meta-analyses, including 8 cohorts and 2 case-control studies. Newcastle-Ottawa Scale results for the cohort studies are described on Supplemental Table 1**,** while descriptions of the case-control studies are reviewed on Supplemental Table 2. Characteristics of the included studies are presented on Supplemental Table 3.

## ART vs natural conception (non-art) (10 studies)

### Risk of cerebral palsy

**Fig. 2:**
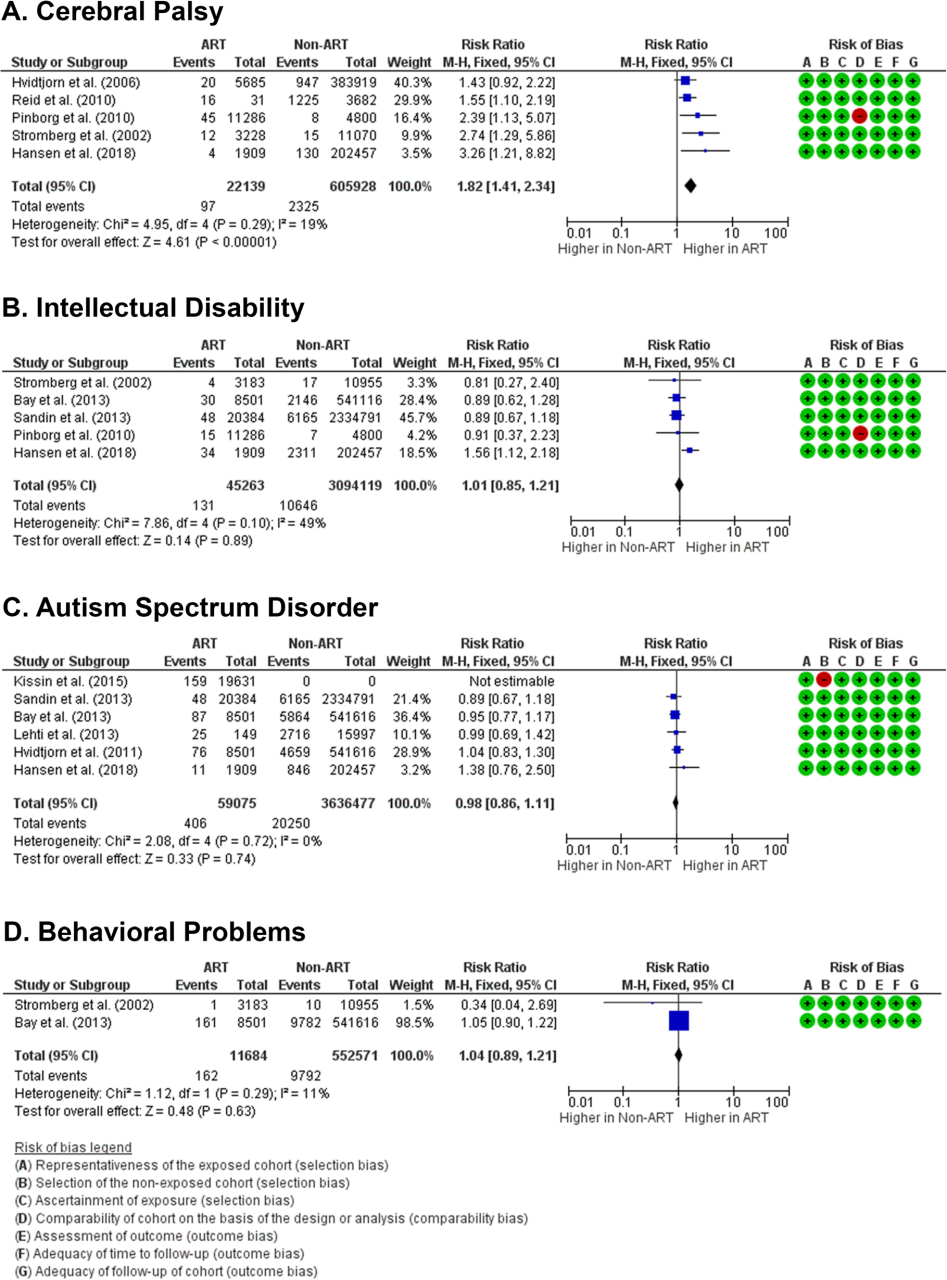
Forest plot of neurodevelopmental disorders in children born from ART compared to children from natural conceptions. (**a**) Based on the reported prevalence of cerebral palsy in children from ART, it can be concluded that children born from ART have a higher risk of acquiring cerebral palsy compared to children from non-ART (natural conceptions). On the other hand, the risk of acquiring intellectual disability (**b**), autism spectrum disorder (**c**), and behavioral problems (**d**) in children from ART is similar with children from natural conceptions

Prevalence of cerebral palsy was reported in 5 studies [21, 22, 26, 27, 29]. ART was associated with higher risk of cerebral palsy (RR 1.82 [1.41, 2.34], *z* = 4.61, *p* < 0.00001; *I*^2^ = 19%, *p* = 0.29) (Fig. 2a), with an absolute risk of 7 per 1000 births at ART group and 4 per 1000 births at baseline population (Supplemental Table 5). No risk of publication bias (Egger’s *p* = 0.501) and no small-study bias (Habord’s *p* = 0.962, Peter’s *p* = 0.991) were acknowledged (Supplemental Table 4).

### Risk of intellectual disability

Risk of intellectual disability (new terminology for mental retardation in DSM-5 and ICD-11) [30] was reported in 5 studies [20, 21, 26, 28, 29]. Risk of intellectual disability in children born after ART treatments was not different from the risk in children from natural conceptions (RR 1.01 [0.85,1.21]; *z* = 0.14, *p* = 0.89; *I*^2^ = 49%, *p* = 0.10) (Fig. 2b), with the same absolute risk of 3 per 1000 births (Supplemental Table 5). Risk of publication bias (Egger’s *p* = 0.754) and small-study bias (Habord’s *p* = 0.872, Peter’s *p* = 0.963) were undetected (Supplemental Table 4).

### Risk of autism spectrum disorder

Six studies reported the risk of ASD [20, 21, 23–25, 28]. One study [24] was excluded as it did not include children from natural conceptions as control. The risk of acquiring ASD in ART children is insignificantly different with its risk in naturally conceived children (RR 0.98 [0.86, 1.11], *z* = 0.33, *p* = 0.74; *I*^2^ = 0%, *p* = 0.72) (Fig. 2c), with identical absolute risk at 6 per 1000 births (Supplemental Table 5). Risk of publication bias (Egger’s *p* = 0.290) and small-study bias (Habord’s *p* = 0.836, Peter’s *p* = 0.506) were unidentified (Supplemental Table 4).

### Behavioral problems

Risk of behavioral problems was reported in 2 studies [20, 29], and the results showed no differences in this risk between ART and naturally conceived group (RR 1.04 [0.89, 1.21], *z* = 0.48, *p* = 0.63; *I*^2^ = 11%, *p* = 0.29) (Fig. 2d). The absolute risk is 19 children per 1000 births at ART group and 18 per 1000 births at naturally conceived group (Supplemental Table 5). Risk of bias and small-study effects could not be estimated due to limited studies (*n* = 2).

Meta-analysis on the overall data showed that ART children have a higher overall risk of neurodevelopmental disorders (RR 1.05 [0.97, 1.14], *z* = 1.29, *p* = 0.20; *I*^2^ = 54%, *p* = 0.005). Heterogeneity was identified for the overall studies, and this might be due to cofounder effects. The overall studies showed no risk of publication bias (Egger’s *p* = 0.501) and no small-study bias (Habord’s *p* = 0.962, Peter’s *p* = 0.991) (Supplemental Table 4). The absolute risk effect showed no increase in number of children born with neurodevelopmental disorders with a baseline population risk of 3 per 1000 births (Supplemental Table 5).

## ICSI vs conventional IVF (5 studies)

### Risk of cerebral palsy

**Fig. 3:**
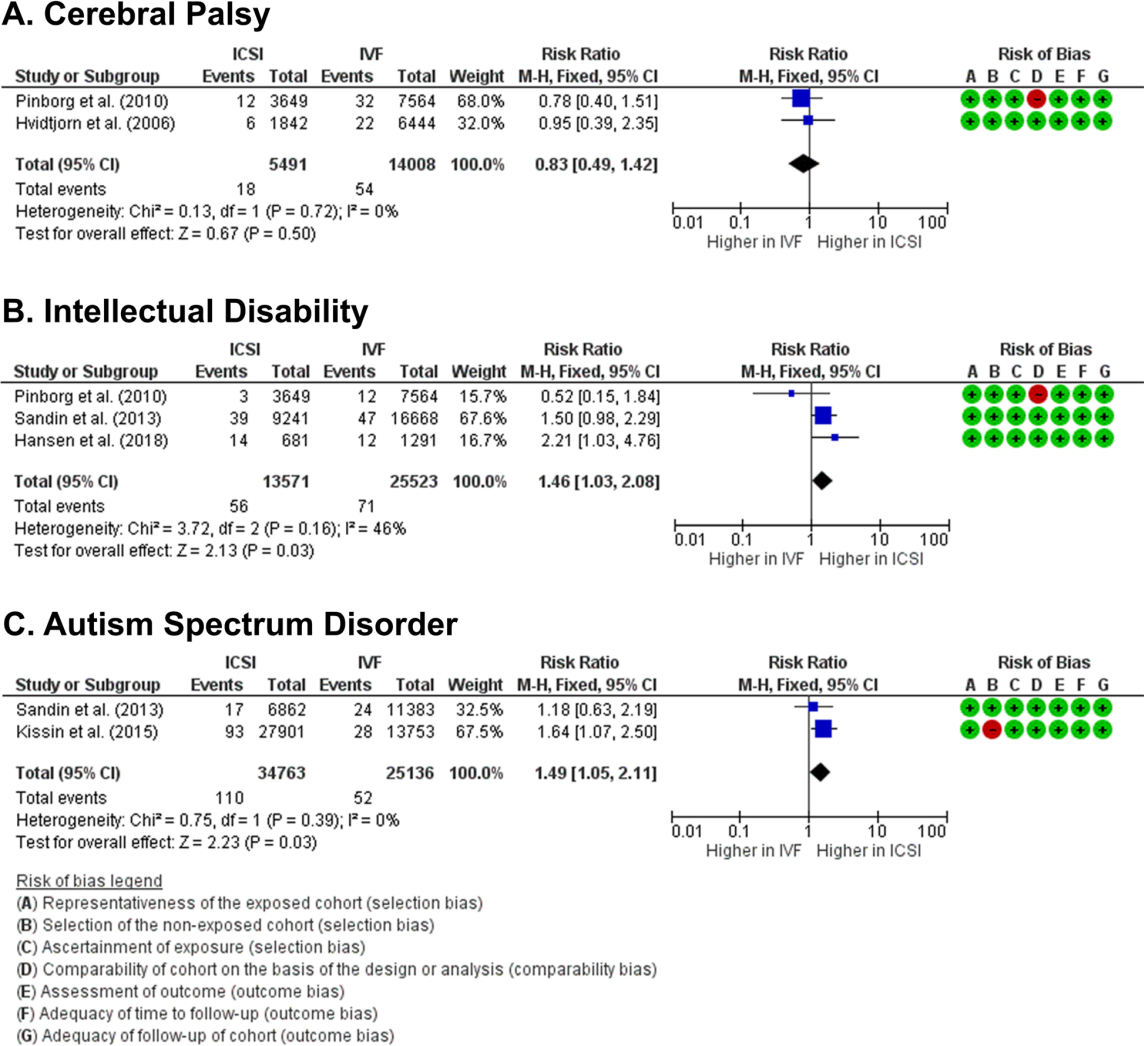
Forest plots of neurodevelopmental disorders in children born from ICSI compared to conventional IVF. Meta-analysis results show that (**a**) risk of cerebral palsy in both groups is similar, while risk of intellectual disability (**b**) and autism spectrum disorder (**c**) in children from ICSI are significantly higher than children from conventional IVF

Prevalence of cerebral palsy in children from ICSI and conventional IVF was reported in 2 studies [22, 26]. ICSI procedure causes no increase in the prevalence of cerebral palsy compared to conventional IVF (RR 0.83 [0.49, 1.42], *z* = 0.67, *p* = 0.50; *I*^2^ = 0%, *p* = 0.72) (Fig. 3a), with an absolute risk 1 fewer children per 1000 birth at ICSI group, while IVF population risk is 4 per 1000 births (Supplemental Table 6). The risk of bias statistic and small-study effects could not be estimated (Supplemental Table 4).

### Risk of intellectual disability

Prevalence of intellectual disability was reported in 3 studies [21, 26, 28]. An increased risk of intellectual disability was identified in the ICSI group compared to conventional IVF (RR 1.46 [1.03, 2.08]; *z* = 2.13, *p* = 0.03; *I*^2^ = 46%, *p* = 0.16) (Fig. 3b), with absolute risk at ICSI group is 5 per 1000 births, while in IVF group is 3 per 1000 births (Supplemental Table 6). No risk of publication bias (Egger’s *p* = 0.487) and small-study bias (Habord’s *p* = 0.786, Peter’s *p* = 0.311) were detected (Supplemental Table 4).

### Risk of autism spectrum disorder

The number of children diagnosed with ASD was reported in 2 studies [24, 28]. There is an increased risk of ASD in children born after ICSI procedure compared to conventional IVF (RR 1.49 [1.05, 2.11], *z* = 2.23, *p* = 0.03; *I*^2^ = 0%, *p* = 0.39) (Fig. 3c), with an absolute risk effect of 1 more children per 1000 births compared to IVF population risk of 2 per 1000 births (Supplemental Table 6). The risk of bias statistic and small-study effects could not be calculated (Supplemental Table 4).

The overall evaluation showed higher prevalence of neurodevelopmental disorders in children from ICSI compared to children from conventional IVF (RR 1.49 [1.05, 2.11], *z* = 2.23, *p* = 0.03; *I*^2^ = 0%, *p* = 0.39).

## Frozen vs fresh embryo transfer (6 studies)

### Risk of cerebral palsy

**Fig. 4:**
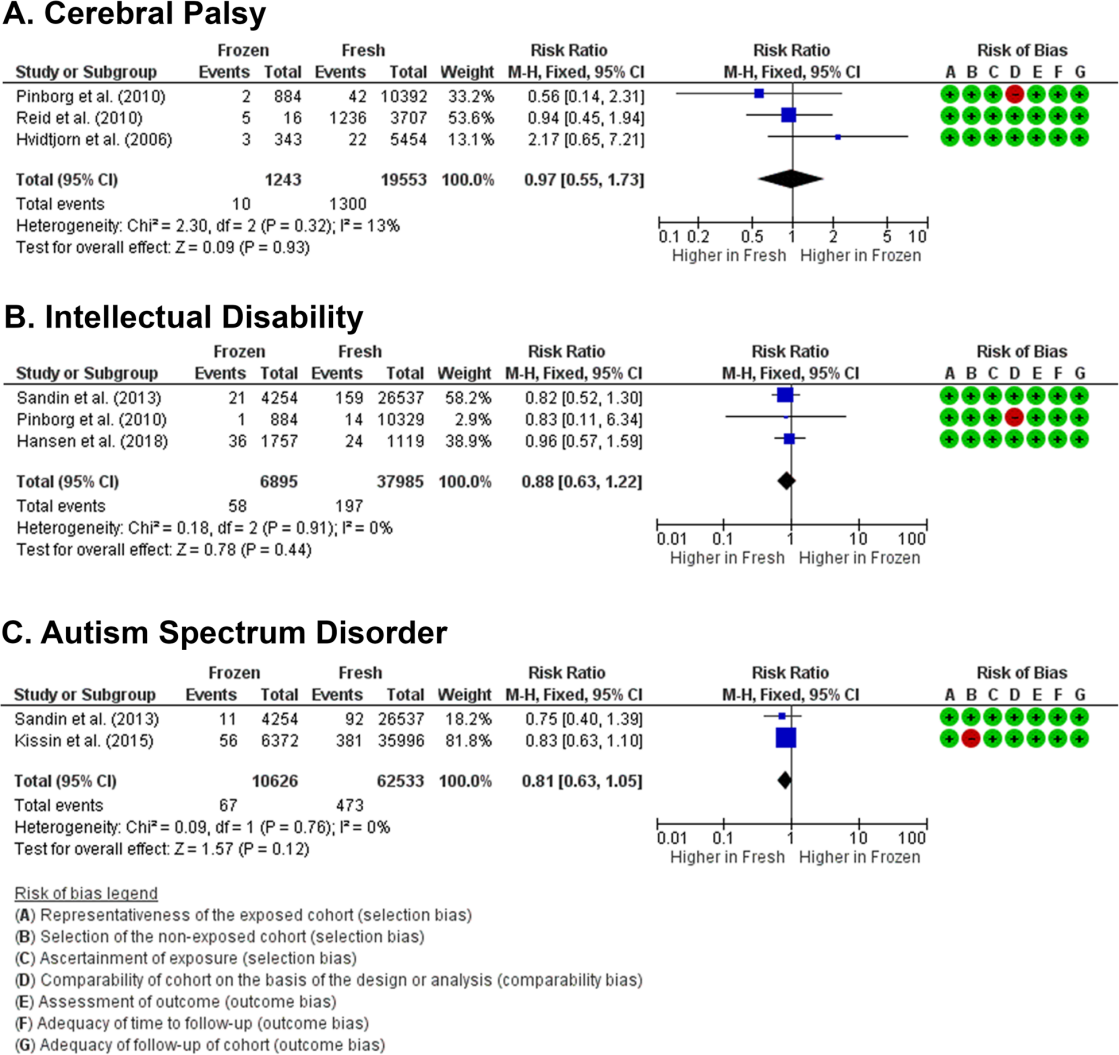
Forest plots of neurodevelopmental disorders in children born from frozen embryo transfer compared to fresh embryo transfer. Results from meta-analysis of published data on neurodevelopmental disorders in children from frozen embryo transfer have similar risks with children born from fresh embryo transfer in acquiring cerebral palsy (**a**), intellectual disability (**b**), and autism spectrum disorder (**c**) at later stages of life

Prevalence of cerebral palsy was reported in 3 studies [22, 26, 27]. Risk of cerebral palsy in children from frozen embryo transfer was similar to fresh embryo transfer (RR 0.97 [0.55, 1.73], *z* = 0.09, *p* = 0.93; *I*^2^ = 13%, *p* = 0.32 (Fig. 4a), with an absolute risk of 64 children in frozen group and 66 children in fresh group per 1000 births or 2 less children at risk compared to fresh group (Supplemental Table 7). The high number of children at risk was due to one study of case-control with 1:2 ratio of children with CP and normal children [27]. Risk of publication bias (Egger’s *p* = 0.974) and small-study bias (Habord’s *p* = 0.601, Peter’s *p* = 0.186) were undetected (Supplemental Table 4).

### Risk of intellectual disability

Prevalence of intellectual disability was reported in 3 studies [21, 26, 28]. Risk of intellectual disability in children born after frozen embryo transfer is not significantly different compared to children from fresh embryo transfer (RR 0.88 [0.63,1.22]; *z* = 0.78, *p* = 0.44; *I*^2^ = 0%, *p* = 0.91) (Fig. 4b), with absolute risk at 4 per 1000 births in frozen embryo transfer group and 5 per 1000 births in fresh embryo transfer group (Supplemental Table 7). Risk of publication bias (Egger’s *p* = 0.619) and small-study bias (Habord’s *p* = 0.937, Peter’s *p* = 0.293) were undetected (Supplemental Table 4).

### Risk of autism spectrum disorder

Risk of ASD was reported in 2 studies [24, 28]. Risk of ASD in children born after frozen embryo transfer is similar to children from fresh embryo transfer (RR 0.81 [0.63, 1.05], *z* = 1.57, *p* = 0.12; *I*^2^ = 0%, *p* = 0.76) (Fig. 4c), with absolute risk at 7 per 1000 births in frozen group and 8 per 1000 birth in fresh group (Supplemental Table 7). The risk of bias statistic and small-study effects could not be estimated due to the limited number of studies (Supplemental Table 4).

Overall analysis showed that the children from frozen embryo transfer do not develop higher risk of neurodevelopmental disorders compared to children from fresh embryo transfer (RR 0.85 [0.70,1.03]; *z* = 1.69, *p* = 0.09; *I*^2^ = 0%, *p* = 0.87). The risk of neurodevelopmental disorder in children from frozen embryos is at 14 per 1000 births while in fresh embryos was 16 per 1000 births (Supplemental Table 7).

## Cofounder effects

**Fig. 5:**
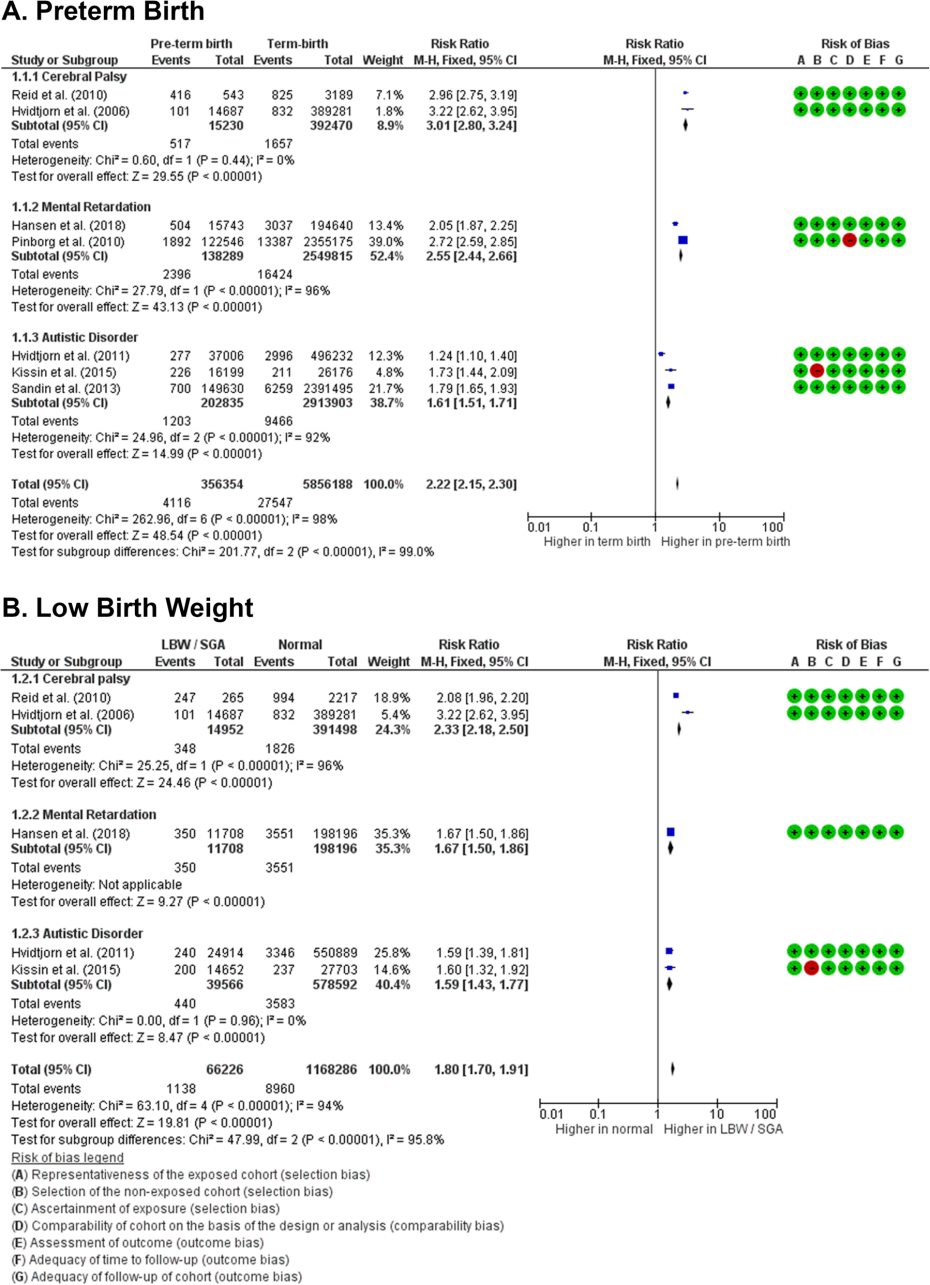
Forest plots of potential cofounders in children from ART. The results of meta-analyses clearly show that children with history of preterm birth (**a**) or low birth body weight (**b**) would also have increased risks of acquiring neurodevelopmental disorders, including cerebral palsy, intellectual disability, and autistic disorders

Additionally, potential cofounders to the results were also evaluated. Two cofounders’ effects were confirmed by meta-analysis, including preterm birth and low birth body weight. The results showed that the risk of neurodevelopmental disorders is higher in children with history of preterm birth (RR 2.22 [2.15, 2.30]; *z* = 48.54, *p* < 0.0001; *I*^2^ = 98%, *p* < 0.0001) (Fig. 5a), with the absolute risk is 6 more children born with neurodevelopmental disorders in every 1000 births (Supplemental Table 8).

Meanwhile, the risk of neurodevelopmental disorders in children with history of low birth body weight (small for gestational age) is also higher compared to children without any history of low birth body weight (RR 1.80 [1.70 to 1.91]; *z* = 19.81, *p* < 0.0001; *I*^2^ = 94%, *p* < 0.0001) (Fig. 5b), with the absolute risk is 6 more children born with neurodevelopmental disorders in every 1000 births (Supplemental Table 8).

ART procedures have the potentials to cause higher rates of multiple birth (RR 14.57 [14.42, 14.73], *z* = 472.11, *p* < 0.0001; *I*^2^ = 100%, *p* < 0.0001), preterm birth (RR 3.83 [3.82, 3.93], *z* = 472.11, *p* < 0.0001; *I*^2^ = 100%, *p* < 0.0001), and low birth body weight (RR 5.66 [5.50, 5.82], *z* = 118.10, *p* < 0.0001; *I*^2^ = 100%, *p* < 0.0001) compared to non-ART procedure (Supplemental Figure 1), with the absolute risk of 386 more multiple birth, 167 more preterm birth, and 219 more low birth body weight per 1000 births (Supplemental Table 9).

## Discussion

To our knowledge, a meta-analysis on the neurodevelopmental outcomes in children from ART has never been performed. This study aimed to make consensual conclusions from all studies that investigated the neurodevelopmental outcomes in children from ART, including ICSI, conventional IVF, frozen embryo transfer, and fresh embryo transfer. The results of this study are expected to provide the reliable information for the patients and healthcare workers to consider before starting ART.

As seen in Fig. 2, without taking specific details of the ART treatments into account, the results show that ART children develop an increased risk of cerebral palsy, but not intellectual disability, ASD, or behavioral problems. Cerebral palsy is a permanent neurological disorder characterized with disability to coordinate muscle tones and movements. This condition is caused by the damage or dysgenesis of brain part(s) that are involved in the coordination and movements of the body. It has been consensually accepted that the common risk factors for cerebral palsy are preterm birth and low birth body weight [31, 32]. In our study, we could confirm that these are the most likely risk factors that were presented among children from ART with cerebral palsy (Fig. 5, Supplemental Figure 1). The ART procedures are associated with higher preterm birth and low birth body weight, as they are also related to higher rates of multiple birth. ART procedures could cause monozygotic twinning related to zona pellucida manipulation and extended in vitro culture. Moreover, double embryo transfers during IVF procedures resulted in higher chance of multiple pregnancy [33]. Since the pathogenesis of cerebral palsy could be initiated with insults during gestation or after delivery, we could not conclude if the pathogenesis of cerebral palsy among ART children is due to the respiratory distress that is caused by immature lungs, which eventually lead to cerebral hypoxia after birth, or as a consequence of dysgenesis of part(s) of the brain that is related to movements and coordination. Nonetheless, these findings revealed the importance of optimum prenatal and neonatal care for ART children.

Interestingly, when the neurodevelopmental outcomes in children from ICSI were compared to the outcomes in children from conventional IVF, increased risks of intellectual disability and ASD were identified in the ICSI group. It is widely known that in some cases, intellectual disability and cerebral palsy are overlapping clinical conditions [34]. However, as the differences in the prevalence of cerebral palsy in children from ICSI and conventional IVF are statistically insignificant, we speculate that in these cases, intellectual disability is more likely due to inert factor(s), such as genetics. Furthermore, ASD has been associated with numerous genetic disorders, and has never been indicated as a result of neonatal insults. Thus, we argue that the cause of higher risks of intellectual disability and ASD in children from ICSI is genetic related.

As we know, ICSI is intended to prevent fertilization failure. One of the indications that becomes practitioners’ common consideration when suggesting ICSI is sperm immotility [35]. Despite successes of achieving pregnancies after ICSI using immotile or morphologically abnormal spermatozoa [36–39], reports have suggested that the rates of DNA fragmentation in immotile spermatozoa are higher than in normal spermatozoa [40]. Furthermore, poor-quality oocytes, advanced maternal age, and unexplained infertility have also been used as indications to perform ICSI [41]. Each of the previously mentioned indication has also been associated with higher rates of DNA fragmentation; hence, this might be related to neurodevelopmental disorders. Study by Porokhovnik et al. correlates infantile autism with elevated DNA damage degree [41]; thus, our findings might be related to any of these factors.

As cryopreservation became more integrated to the common ART practice nowadays, the comparison between children from frozen embryo transfer with children from fresh embryo transfer was also performed. As previous reports have suggested, the risk of acquiring DNA fragmentations during cryopreservation and thawing processes might be associated with reduced numbers and proportion of trophectoderm cells [42], higher prevalence of neurodevelopmental disorders in children from frozen embryo transfer is expected. To our surprise, the meta-analysis results showed that there are no significant differences between the risks of neurodevelopmental disorders in children from frozen embryo transfer compared to the risk in children from fresh embryo transfer. Previous studies also reported that frozen embryo transfer resulted in increased live birth rate and higher neonatal birth body weight [43–45]. The improvement of cryopreservation techniques, such as vitrification and elective frozen embryo transfer, might result in better pregnancy and neonatal outcome [42, 46].

### Limitations

The present study has several limitations. First, the number of studies that reported neurodevelopmental outcomes from different ART procedures is limited. This limitation correlates with the limited number of patients and the possibilities that some of the findings might be population specific. Secondly, singleton and multiple births could not be differentiated in some studies. Previous studies suggested that multiple pregnancy carries higher risks of prematurity and low birth body weight [47, 48], both could potentially contribute to the risks of neurodevelopmental disorders [49, 50]. Third, the matched case-control studies could introduce potential conclusion bias on the absolute number of children at risk in the normal population. To overcome all of these limitations, future studies that involve larger populations with sufficient background information are required.

## Conclusion

In conclusion, children from ART have a higher risk of acquiring cerebral palsy than children from natural conceptions. This increased risk is possibly associated with confounding factors, including preterm birth and low body birth weight. Based on this finding, it is reasonable to perform deliveries of children from ART at facilities with available neonatal intensive care unit (NICU); thus, treatment of potential cerebral insults after birth can be optimized. Furthermore, children from ICSI carry higher risks of intellectual disability and ASD than children from conventional IVF. These findings suggest the importance of long-term follow-up and preimplantation genetic screening, particularly in fertilizations by ICSI. Lastly, cryopreservation and thawing of embryos did not seem to cause increases in any risks of neurodevelopmental disorders; therefore, freeze-all strategy seemed plausible.

## Supplementary Information

**Supplemental Table 1. **Newcastle Ottawa Scale on Cohort Studies. **Supplemental Table 2**. Newcastle-Ottawa Scale on Case-Control Studies. **Supplemental Table 3**. Characteristics of the study. **Supplemental Table 4**. Statistical Summary of Meta-Analysis. **Supplemental Table 5**. Summary of findings and study quality assessment with GRADE approach of ART vs Non-ART studies. **Supplemental Table 6**. Summary of findings and study quality assessment with GRADE approach of ICSI vs IVF studies. **Supplemental Table 7**. Summary of findings and study quality assessment with GRADE approach of Frozen vs Fresh Embryo Transfer studies. **Supplemental Table 8**. Summary of findings and study quality assessment with GRADE approach of Confounder Effects studies. **Supplemental Table 9**. Summary of findings and study quality assessment with GRADE approach of Risk of Multiple Birth, Preterm Birth, Low birth body weight in ART and Non-ART children.

## Data Availability

All data generated or analyzed during this study are included in this published article and its supplementary information files.

## Abbreviations

ART: Assisted reproductive treatment
CP: Cerebral palsy
IVF: In vitro fertilization
ICSI: Intracytoplasmic sperm injection
LBW: Low birth body weight
PT: Preterm birth
SGA: Small for gestational age
