# Supplementary material for: Risk of neurodevelopmental disorders in children born from different ART treatments: a systematic review and meta-analysis
Source: J Neurodev Disord. 2020 Dec 13;12:33. doi: 10.1186/s11689-020-09347-w (PMC7734782; doi:10.1186/s11689-020-09347-w)
Supplement: Supplementary file 1 — Supplemental Table 1. Newcastle Ottawa Scale on Cohort Studies. Supplemental Table 2. Newcastle-Ottawa Scale on Case-Control Studies. Supplemental Table 3. Characteristics of the study. Supplemental Table 4. Statistical Summary of Meta-Analysis. Supplemental Table 5. Summary of findings and study quality assessment with GRADE approach of ART vs Non-ART studies. Supplemental Table 6. Summary of findings and study quality assessment with GRADE approach of ICSI vs IVF studies. Supplemental Table 7. Summary of findings and study quality assessment with GRADE approach of Frozen vs Fresh Embryo Transfer studies. Supplemental Table 8. Summary of findings and study quality assessment with GRADE approach of Confounder Effects studies. Supplemental Table 9. Summary of findings and study quality assessment with GRADE approach of Risk of Multiple Birth, Preterm Birth, Low birth body weight in ART and Non-ART children. [file 11689_2020_9347_MOESM1_ESM.docx]

**Supplemental Table 1.** Newcastle Ottawa Scale on Cohort Studies

| **Name of Study (Year)** | **Selection** | | | | **Comparability** | **Outcome** | | | **Total Score** |
| --- | --- | --- | --- | --- | --- | --- | --- | --- | --- |
|  | Representativeness of the exposed cohort  (a) truly representative of the average IVF treated subjects in the community*  (b) somewhat representative for the IVF treated subjects in the community*  (c) selected group of users  (d) no description of the derivation of the cohort | Selection of the non-exposed cohort  (a) drawn from the same community as the exposed cohort*  (b) drawn from different source  (c) no description of the derivation of the non-exposed cohort | Ascertainment of exposure  (a) secure record*  (b) structured interview*  c) written self report  (d) no description | Demonstration that outcome of interest was not present at start of study  (a) yes*  (b) no | Comparability of cohort on the basis of the design or analysis  (a) study controls for maternal age, birthweight, gestational age*  (b) study controls for any additional factors* | Assessment of outcome  (a) Independent blind assessment*  (b) Record linkage*  (c) Self-report  (d) No description | Was follow-up long enough for outcomes to occur  (a) yes (3-year minium follow-up)* (b) no | Adequacy of follow-up of cohort  (a) complete follow-up, all subjects accounted for*  (b) subjects lost to follow-up are unlikely to introduce bias – small number lost <20%  (c) follow-up rate <80% and no description of those lost  (d) no description *or unclear* |  |
| [Bay et al. (2013)](file:///C:\Users\samgr\AppData\Roaming\Microsoft\Word\Bay%20et%20al.%20(2013)) | (a) Data from Danish medical birth register, Denmark and Danish IVF Register, Danish national Prescription Register, Danish Psychiatric Central Research Register* | (a) Data of the naturally conceived children were drawn from the National Register* | (a) Secure record from National Register* | (a) Intellectual Disability , psychiatric, and behavioral disorders not present at time of enrolling (birth)* | (a,b) Adjusted for maternal age, parity, educational level, smoking in pregnancy, maternal psychiatric history, birth year, child’s sex, and multiplicity** | (b) Record linkage from national record registry* | (a) Yes, the follow-up time were 8-17 years* | (d) No statement | 8 |
| Belva et al. (2007)a | (c) Children at 8 years of age born in VU Brussel Center of Reproductive Medicine | (b) Normally conceived children recruited from normal schools | (a) Secure record from hospital records* | (a) Behavioral problems were not detected at time of enrolling (birth)* | Maternal age, birth weight, and other confounders are not matched in the design nor adjusted for in the statistical analysis (not provided) | (c) Socio- demographic, maternal complication, neonatal medical history were obtained through self-reported questionnaire | (a) Follow up were done since birth until 8 years of age* | (a) complete data for the participants* | 4 |
| Beydoun et al. (2010)a | (c) Young adults conceived by IVF at the Jones Institute for Reproductive Medicine (JIRM), the Division of Reproductive Endocrinology and Infertility at Eastern Virginia Medical School (EVMS) in Norfolk, Virginia | (b) Respondents were compared on selected characteristics to a sub-sample of the National Health and Nutrition Examination Survey (NHANES) | (a) Secure records from the hospital of IVF-conceived young adults* | (a) Chronic diseases and psychiatric disorders were not present at the time of enrolling (birth)* | (b) Only gender are adjusted in the statistical analysis* | (c) 90 questions on self-report questionnaire | (a) Yes follow up were done at mean 21.2 years of age | (a) complete data for all the participants* | 5 |
| D'Souza et al. (1997)a | (c) Children born at St. Mary’s Hospital, Manchester, UK | (a) Naturally conceived children born at St. Mary's Hospital, UK* | (a) Hospital records* | (a) Congenital anomalies and disabilities were not present at time of enrolling (birth)* | Maternal age, birth weight, and other confounders are not matched in the design nor adjusted for in the statistical analysis (not provided) | (a) General medical examination*  (c) Self-reported medical history | (a) Yes, follow up for 4 years after birth* | (d) No description | 5 |
| [Hansen et al. (2018)](file:///C:\Users\samgr\AppData\Roaming\Microsoft\Word\Hansen%20et%20al.%20(2018)) | (a) Total live birth from 1994-2002 in Western Australia* | (a) Naturally conceived children born in 1994-2002 in Western Australia* | (a) Regional register record* | (a) Congenital anomalies, Intellectual Disability , intellectual disabilities were not present at birth* | (a,b) Risk ratio were adjusted for sex, y of birth group, parity group, maternal age group, delivery mode, PHI, and marital status** | (b) Record linkage* | (a) Yes, follow up for 8 years | (d) No description | 8 |
| [Hvidtjorn et al. (2006)](file:///C:\Users\samgr\AppData\Roaming\Microsoft\Word\Hvidtjorn%20et%20al.%20(2006)) | (a) Data from Danish Medical Birth Register cross-linked with National Register of Hospital Discharges, and Statistics Denmark* | (a) Data of the naturally conceived children obtained from Danish Medical Birth Register cross-linked with National Register of Hospital Discharges, and Statistics Denmark* | (a) National registry record* | (a) Cerebral palsy were not detected at time of enrolling (birth)* | (a,b) Hazard risk ratio adjusted for maternal educational level, age, parity, gender, multiplicity, and preterm delivery ** | (b) Record linkage* | (a) Yes, follow up for 1 -7 years of age* | (d) No description | 8 |
| [Hvidtjorn et al. (2011)](file:///C:\Users\samgr\AppData\Roaming\Microsoft\Word\Hvidtjorn%20et%20al.%20(2011)) | (a) Data from Danish medical birth register cross-linked with IVF Register* | (a) Data from naturally conceived children from the sam register* | (a) National registry record* | (a) Risk of autism were not detected at time of birth* | (a,b) Adjusted for maternal age, educational level, parity, smoking, body weight and multiplicity** | (b) Record linkage from Danish Psychiatric Central Register according to ICD-10* | (a) yes, follow up for 5-13 years* | (d) No description | 8 |
| [Kissin et al. (2015)](file:///C:\Users\samgr\AppData\Roaming\Microsoft\Word\Kissin%20et%20al.%20(2015)) | (a) Data from national ART Surveillance System 1996-2006, California Birth Certificate 1997-2006, California Department of Developmental Services Autism Caseload 1997-2011* | (c) No controls from naturally conceived chidlren available | (a) National registry record* | (a) ASD were not detected at time of enrolling (birth). The date of autism diagnosis was defined as the date of enrollment on the DDS Autism Caseload database. Since the majority of autism cases are typically diagnosed by age 5* | (a,b) Hazard risk ratio were adjusted for infant sex (male, female), gestational age (≥37 weeks,,37 weeks), birthweight (≥2500 g,,2500 g), maternal and paternal age at delivery (,35 years, 35 –39 years, ≥40 years), number of previous births (0,≥1), mode of delivery (vaginal, Cesarean), and birth year** | (b) Record linkage* | (a) yes, follow up until 5 years of age* | (d) No description | 7 |
| Knoester et al. (2007)a | (c) Children born in Leiden University Medical Center | (b) Naturally conceived children from 16 different schools | (a) Hospital secure record* | (a) ASD and other psychiatric disorders were not reported at time of enrollment (birth)* | Maternal age, birth weight, and other confounders are not matched in the design nor adjusted for in the statistical analysis (not provided) for neurodevelopmental outcome | (c) Neurological outcome were self-reported from questionnaire | (a) Yes, follow-up 5-8 years old* | (d) No description | 3 |
| Middleburg et al. (2009)a | (c) Department of Reproductive Medicine University Medical Center Groningen, Netherlands | (a) Naturally conceived couple with parental characteristics, such as parity and age, of this cohort would resemble the characteristics of IVF couples* | (a) Hospital secure record* | (a) ASD and other neurological outcomes were not present at time of enrollment (birth)* | (a,b) Adjusted for gestational age, birthweight, vanishing twins and time to pregnancy** | (a) Independent blind assessment with Touwen and Hempel Neurological Examination* | (b)No, follow up was shorter than 3 years (4,10,and 18 months post partum) | (b)  Neurological assessment at 18 month:  2 COH-IVF and 1 MNC-IVF and 3 NC lost to follow up* | 7 |
| Pinborg et al. (2003)a | (a) Data from Danish National birth register* | (c) No naturally conceived control for singleton | (a) Secure national register record* | (a) Neurological sequelae were not present at time of birth* | (a) Speech therapy, special needs, and general helath were adjusted for birthweight* | (c) Self-reported questionnaire | (a) Yes, children were aged 3 to 4 years at time of follow-up* | (d) No description | 5 |
| Pinborg et al. (2004)a | (a) Data from Danish National birth register* | (c) No naturally conceived control for singleton | (a) Secure national register record* | (a) Neurological sequelae were not present at time of birth* | (b) Adjusted for sex and year of birth* | (b) Record linkage* | (a) Yes, children were aged 2 to 7 years at time of follow-up* | (d) No description | 6 |
| [Pinborg et al. (2010)](file:///C:\Users\samgr\AppData\Roaming\Microsoft\Word\Pinborg%20et%20al.%20(2010)) | (a) Data from The Danish national Cohort study 1995-2006* | (a) Fresh embryo transfer and naturally conceived children with data from The Danish national Cohort study 1995-2006* | (a) National registry based record* | (a) neurological, Intellectual Disability , and cerebral palsy were not present at time of enrollment (birth)* | (a,b) Multiple logistic regression analyses were performed with adjustment for maternal age, parity, child year of birth and child gender** | (b) Record linkage from national database* | (a) Yes, follow-up was done for children aged 12 months until 13 years of age* | (d) No description | 8 |
| [Sandin et al. (2013)](file:///C:\Users\samgr\AppData\Roaming\Microsoft\Word\Sandin%20et%20al.%20(2013)) | (a) Data from Swedish national health register 1982-2007* | (a) Naturally conceived children from Swedish national health register 1982-2007* | (a) National registry based record* | (a) Autism disorder and Intellectual Disability were not present at time of enrollment (birth)* | (a,b) Adjusted for Sex, attained age, birth year, paternal age categorically, maternal age categorically, maternal psychiatric history at offspring birth (yes or no), paternal psychiatric history at offspring birth (yes or no)** | (b) Record linkage from national database* | (a) Yes, follow-up was done for children aged 2 until adults 27 years old* | (d) No description | 8 |
| [Stromberg et al. (2002)](file:///C:\Users\samgr\AppData\Roaming\Microsoft\Word\Stromberg%20et%20al.%20(2002)) | (a) Data from The National Board of Health and Welfare, Sweden 1982-1995* | (a) Naturally conceived children with data from The National Board of Health and Welfare, Sweden* | (a) National registry based record* | (a) Neurological disorder were not present at time of enrollment (birth)* | (a,b) Adjusted for year of birth, birth hospital, sex. Logistic regression analysis showing independent effects of in-vitro fertilisation (IVF), maternal age, male sex, and low birthweight (upper panel) or low gestational age (lower panel) on the risk of needing contact with a childhood disability centre, risk of cerebral palsy, and risk of suspected developmental delay** | (b) Record linkage from national database* | (a) Yes, children aged 18 months to 15 years old were included in the follow-up study* | (d) No description | 8 |

**Supplemental Table 2.** Newcastle-Ottawa Scale on Case-Control Studies

| **Name of Study (Year)** | **Selection** | | | | **Comparability** | **Exposure** | | | **Additional** | **Total Score** |
| --- | --- | --- | --- | --- | --- | --- | --- | --- | --- | --- |
|  | Is the case definition adequate?  (a) Yes, with independent validation* (b) Yes, (e.g. record linkage or based on self reports) (c) No description | Representativeness of the cases  (a) Consecutive or obviously representative series of cases* (b) Potential for selection biases or not stated | Selection of Controls  (a) Community controls* (b) Hospital controls (c) No description | Definition of Controls  (a) No history of disease* (end-point) (b) No description of source | Comparability of the cases and controls on the basis of the design or analysis  (a) Study controls formaternal age* (b) Study controls for any additional factors* | Ascertainment of exposure  (a) Secure record (e.g. surgical record)* (b) Structured interview blind to case/control (c) Interview not blinded to case/control (d) Written self report or medical record only (e) No description | Same method of ascertainment (a) Yes* (b) No | Non-response rate (a) Same rate for both groups* (b) Non-respondents described (c) Rate different and no designation | mode of conception and embryo transfer method stated?  (a) Mode of conception (ICSI, IVF, etc.) stated*  (b) Singleton vs twins  (c) ET method (fresh/frozen stated)*  (c) No description |  |
| [Lehti et al. (2013)](file:///C:\Users\samgr\AppData\Roaming\Microsoft\Word\Lehti%20et%20al.%20(2013)) | (b) Cases identified through record linkage, diagnosed according to ICD 9/10 | (a) All eligible cases over a defined time/catchment area* | (a) Controls derived from the same registry and would be cases if had outcome* | (a) No history of ASD* | (a,b)Maternal age and other confounders are adjusted in the statistical analysis** | (a) Secure record(medical birth register)* | (a) Yes* | (a) Same rate can be assumed* | (b) Singleton vs twins* | 8(1) |
| [Reid et al. (2010)](file:///C:\Users\samgr\AppData\Roaming\Microsoft\Word\Reid%20et%20al.%20(2010)) | (b) Cases identified through record linkage | (a) All eligible cases over a defined time/ catchment area* | (a) Controls derived from the same registry and would be cases if had outcome* | (a) No history of cerebral palsy* | (a,b) Maternal age, Sex, spontaneous abortions, parity, SGA, and birthweight are adjusted in the statisticl analysis* | (a) Secure record (medical birth register)* | (a) Yes* | (a) Same rate can be assumed* | (a) ART vs non ART*  (c) Fresh/frozen*  (b) Singleton only* | 8(3) |

**Supplemental Table 3.** Characteristics of the study

| **Author, Country, and timeframe** | **Exclusion criteria** | **Study design** | **Exposure** | **Type of Neurodevelopmental Disorder** | **Comparison group** | **Cofounders adjusted** | **Outcome summary** |
| --- | --- | --- | --- | --- | --- | --- | --- |
| [Bay et al. (2013)](file:///C:\Users\samgr\AppData\Roaming\Microsoft\Word\Bay%20et%20al.%20(2013))  Denmark  Jan 1995 - 31 Dec 2003 | NS | National record linkage Cohort study | IVF/ICSI (14,991)  OI/IUI (18,148) | Any mental disorder (F70.0-F99.9); any mental retardation and degrees of Intellectual Disability (F70.0-F79.9); developmental disorders, all (F80.0-F89.9); developmental disorders of speech and language (F80x); scholastic skills (reading, spelling or calculating disorders) (F81x) or motor function (F82x); developmental disorders, mixed (F83x), pervasive developmental disorders (Autism Spectrum Disorder) (F84x); developmental disorders, other (F88.0-F89.9); behavioural and emotional disorders, all (F90.0-F98.8); hyperkinetic disorder (F90x); conduct, emotional or social disorder (F91.0-F94.9); tic disorders (F950-F95.9); behavioural and emotional disorders, other (F98.8), and mental disorders, other (F99x) | Spontaneous conception  (555,828) | + maternal age  + parity  + educational level  + smoking in pregnancy  + maternal psychiatric history  + birth year  + child’s sex  + multiplicity | The risk of mental disorders in children born after in vitro fertilisation or intracytoplasmic sperm injection was low, and was no higher than in spontaneously conceived children, except for a borderline significant increased risk of tic disorders (hazard ratio 1.40, 95% confidence interval 1.01 to 1.95; absolute risk 0.3%). |
| [Hansen et al. (2018)](file:///C:\Users\samgr\AppData\Roaming\Microsoft\Word\Hansen%20et%20al.%20(2018))  Western Australia  1994-2002 | NS | National record linkage cohort study | IVF  -Singleton (1291)  -Multiple (648)  ICSI  -Singleton (618)  -Multiple (319)  Fresh ET (1,757)  Frozen ET (1,119) | Intellectual Disability with inclusion criteria: IQ<70, and deficits in adaptive functioning with evidence of developmental delay before 18 year of age  Diagnosis was done using the American Association of Intellectual Disability classification system | Non-ART born children  -Singleton (202,457)  - Multiple (5,294) | sex, y of birth group, parity group, maternal age group, delivery mode, PHI, and marital status | Children conceived using ART had a small increased risk of ID (risk ratio 1.58; 95% confidence interval 1.19–2.11) even when analyses were restricted to singleton births (risk ratio 1.56; 95% confidence interval 1.10–2.21). The risk of ID was more than doubled for those born very preterm, for severe ID, and after intracytoplasmic sperm injection (ICSI) treatments. Children conceived using ICSI had a greater risk of ID than those conceived using in vitro fertilization |
| [Hvidtjorn et al. (2006)](file:///C:\Users\samgr\AppData\Roaming\Microsoft\Word\Hvidtjorn%20et%20al.%20(2006))  Denmark  Jan 1995 - Dec 2000 | NS | Danish national register, Danish Medical birth register, IVF register, National register of Hospital Discharge | IVF  + Singelton (5,685)  + Twin (3,570)  Contentional IVF (6,444)  ICSI (1,842)  Fresh embryo (8,878)  Frozen embryo (560) | CP was defined with anInternational Classification of Diseases, 10th Revision, code of G80.0 to G83.9 in the NRHD | Non-IVF children  + Singleton (383,919)  + Non-IVF twin (10,794) | gender, parity, maternal age, and educational level | Children born after in vitro fertilization had an increased risk of cerebral palsy; these results were largely unchanged after adjustment for maternal age, gender, parity, small-for-gestational age status, and educational level. The independent effect of in vitro fertilization vanished after additional adjustment for multiplicity or preterm delivery. When both multiplicity and preterm delivery were included in the multivariate models, preterm delivery remained associated strongly with the risk of cerebral palsy. |
| [Hvidtjorn et al. (2011)](file:///C:\Users\samgr\AppData\Roaming\Microsoft\Word\Hvidtjorn%20et%20al.%20(2011))  Denmark  1995-2003 | Mother aged< 20 years | Danish national register, Danish medical birth register, IVF register, Danish drug prescription register, Danish psychiatric central register | Assisted conception  + Singleton (23,483)  + Twins (9,120)  + Triplets (495)  + quadruplet (17)  OI  + Singletons (8,501)  + Twins (6,234)  Triplets (239)  + Quadruplets (5)  IVF  + Singletons (14,982) + Twins (2886)  + Triplets (256)  + Quadruplets (12) | Children with a diagnosis of ASD (F84.0, F84.1, F84.5, F84.8 and F84.9) or, specifically, infantile autism (F84.0) | Natural conception  + Sing (541,616)  + Twins (13,376)  + Triplets (251)  + quadruplets (4) | BW, gestational age, multiplicity, sex, maternal age, educational level, smoking and parity. | In crude analyses, children born after assisted conception had an increased risk of a diagnosis of ASD: crude hazard rate ratio (HRR) 1.25 (95% CI 1.09 to 1.43). In analyses adjusting for maternal age, educational level, parity, smoking, birth weight and multiplicity, the risk disappeared: adjusted HRR 1.13. (95% CI 0.97 to 1.31). |
| [Kissin et al. (2015)](file:///C:\Users\samgr\AppData\Roaming\Microsoft\Word\Kissin%20et%20al.%20(2015))  California  1997-2006 | NS | California Birth certificate, California Department of Developmental Services (DDS) Autism Caseload | IVF, Gamette intrafalopian transfer, Zygote intrafalopian transfer, ICSI surgery or ejaculated, Fresh, frozen embryo transfer  Singleton (19,790)  Twin (19,772)  Triplet (2,821) | Autism Spectrum Disorder is defined by the American Psychiatric Association’s Diagnostic and Statistical Manual, 4th edition (DSM-IV, 1994, revised in 2000), which provides standards for the diagnosis and classification of ASD (APA, 2000). The DDS Autism Caseload database includes children diagnosed under the DSM-IV code 299.0, which includes Autism Spectrum Disorder (hereafter, autism). | No control | Infant and parental characteristics that were adjusted for included infant sex (male, female), gestational age (≥37 weeks,,37 weeks), birthweight (≥2500 g,,2500 g), maternal and paternal age at delivery (,35 years, 35–39 years,≥40 years), number of previous births (0,≥1), mode of delivery (vaginal, Cesarean), and birth year | Among ART-conceived singletons born in California between 1997 and 2006, the incidence of autism diagnosis remained at0.8% (Pfor trend 0.19) and was lower with parental diagnosis of unexplained infertility (adjusted hazard risk ratio [aHRR]; 95% confidence interval: 0.38; 0.15–0.94) and higher when ICSI was used (aHRR 1.65; 1.08–2.52), when compared with cases without these patient and treatment characteristics. Among ART-conceived multiples, the incidence of autism diagnosis between 1997 and 2006 remained at1.2% (Pfor trend 0.93) and was lower with parental diagnosis of tubal factor infertility (aHRR 0.56; 0.35–0.90) and higher when ICSI was used (aHRR 1.71; 1.10–2.66). |
| [Lehti et al. (2013)](file:///C:\Users\samgr\AppData\Roaming\Microsoft\Word\Lehti%20et%20al.%20(2013))  Finland  1991-2005 | + Triplet and higher order pregnancy  + ASDs or severe/profound Intellectual Disability according to the FHDR | Finnish National Birth register and Finnish National Hospital Discharge | Different subtypes of IVF could not be specified  +Singleton (46/149)  + whole (63/229) | All diagnostic codes indicating an ASD were collected according to the ICD-9 in 1991 – 1995 and ICD-10 in 1996 –2007  ASDs (F84) and three of its subcategories: childhood autism (F84.0), Asperger’s syndrome (F84.5) and other pervasive developmental disorder (PDD) and PDD, not otherwise specified (PDD NOS) (F84.8 and F84.9) | Non-IVF  + Singleton (4007/15997)  + Whole sample (4101/16353) | Maternal age, mother’s socioeconomic status (SES), gestational age and parity | No significant association was found between IVF and ASDs (adjusted odds ratio (OR): 0.9, 95% confidence interval (CI): 0.7– 1.3) or its subtypes childhood autism (OR: 0.8, 95% CI: 0.4 –1.5), Asperger’s syndrome (OR: 0.9, 95% CI: 0.5– 1.6) or other pervasive developmental disorder (OR: 1.0, 95% CI: 0.6 –1.6). When only singletons were included, there was an association between IVF and Asperger’s syndrome in an unadjusted analysis (OR: 2.0, 95% CI: 1.1 –3.5) but this was not significant when adjusted for mother’s socioeconomic status or parity. When the analyses were conducted separately for boys and girls, there was a significant association between IVF and Asperger’s syndrome for boys in an unadjusted analysis (OR: 2.1, 95% CI: 1.2 –3.7) but this was not significant in the final adjusted model |
| [Pinborg et al. (2010)](file:///C:\Users\samgr\AppData\Roaming\Microsoft\Word\Pinborg%20et%20al.%20(2010))  Denmark 1995-2007 | NS | Danish IVF register, Danish Medical birth register  National hospital discharge register | IVF (7,564)  ICSI (3,669)  Cryo (957)  Fresh (10,329) | All diagnoses including malignancies were classified according to the International Classification of Diseases, 10 Edition (ICD-10 codes) in the HDR  Neurological (DF70-79+ DG80-83)  Intellectual Disability (DF70-79) cerebral palsy (DG80-83) | Spontaneously conceived  (4,800) | Multiple logistic regression analyses were performed with adjustment for maternal age, parity, child year of birth and child gender | Birth weight was higher in Cryo (mean¼3578 g, SD¼625) versus fresh (mean¼3373 g, SD¼648) and in Cryo versus non-ART (mean¼3537 g, SD¼572), and this was also the case for first birth only. Lower adjusted risk of LBW (odds ratio [OR]¼0.63; 95% confidence interval [CI], 0.45–0.87) and PTB (OR¼0.70; 95% CI, 0.53–0.92) was observed in Cryo versus fresh. Similar LBWand PTB rates were observed when comparing Cryo with non-ART, but the perinatal mortality rate was doubled in Cryo (1.6%) compared with non-ART (0.8%) singletons, and the adjusted risks of very preterm birth (<34 weeks) and neonatal admittance were also significantly increased. No significant differences in the prevalence rates of birth defects, neurological sequelae, malignancies, and imprinting-related diseases were observed between the Cryo and the two control groups. However higher malformation and cerebral palsy rates were observed in the total Fresh vs. non-ART group |
| [Reid et al. (2010)](file:///C:\Users\samgr\AppData\Roaming\Microsoft\Word\Reid%20et%20al.%20(2010))  Australia  1991-2004 | multiple birth (n=174), postneonatal timing (n=104), or no registration number (n=45) | Victorian cerebral Palsy Register, Victoria Perinatal Data collection, | ART (IVF, ICSI, gamete intrafallopian transfer) (16/25) | Cerebral palsy | Naturally conceived  (1225/2457) | spontaneous abortions, parity, Small for gestational age, birthweight, sex | Sixteen (1.3⁄100) of the children with CP and 25 (1.0⁄100) of 2482 children without CP were conceived using ART. There was no significant increase in the odds of children with CP being conceived using ART (adjusted odds ratio 1.19, 95% confidence interval (CI) 0.63, 2.24) nor in the odds of them being conceived by a subfertile couple without ART (adjusted odds ratio 2.7, 95% CI 0.87, 8.36). |
| [Sandin et al. (2013)](file:///C:\Users\samgr\AppData\Roaming\Microsoft\Word\Sandin%20et%20al.%20(2013))  Sweden  1982-2007 | NS | Medical Birth Register, Multi-generation Register, Patient Register, and IVF Register | IVF + fresh (16,668)  + Frozen (2,777)  ICSI  + Fresh (9,241)  + Frozen (1,477)  Surgical/ ejaculated sperm  +Fresh (628) + Frozen (168) | The International Classification of Diseases(ICD) ninth and tenth revisions were used. Focused on Intellectual Disability  and on the narrow diagnosis of infantile and childhood autism (diagnostic codesICD-9 299A orICD-10 F84.0) and do not include other forms of Autism Spectrum Disorder | Spontaneously conceived  (2,510,166) | All models were adjusted for sex, attained age, birth year, paternal age categorically, maternal age categorically, maternal psychiatric history at offspring birth (yes or no), paternal psychiatric history at offspring birth (yes or no), except for “crude,” which was adjusted for sex, attained age, and birth year | Compared with IVF without ICSI with fresh embryo transfer, there were statistically significantly increased risks of Autism Spectrum Disorder following ICSI using surgically extracted sperm and fresh embryos (RR, 4.60 [95% CI, 2.14-9.88]; 135.7 vs 29.3 per 100 000 person-years); for Intellectual Disability following ICSI using surgically extracted sperm and fresh embryos (RR, 2.35 [95% CI, 1.01-5.45]; 144.1 vs 60.8 per 100 000 person-years); and following ICSI using ejaculated sperm and fresh embryos (RR, 1.47 [95% CI, 1.03-2.09]; 90.6 vs 60.8 per 100 000 person-years). When restricting the analysis to singletons, the risks of Autism Spectrum Disorder associated with ICSI using surgically extracted sperm were not statistically significant, but the risks associated with ICSI using frozen embryos were significant for Intellectual Disability (with frozen embryos, RR, 2.36 [95% CI, 1.04-5.36], 118.4 vs 50.6 per 100 000 person-years]; with fresh embryos, RR, 1.60 [95% CI, 1.00-2.57], 80.0 vs 50.6 per 100 000 person-years). |
| [Stromberg et al. (2002)](file:///C:\Users\samgr\AppData\Roaming\Microsoft\Word\Stromberg%20et%20al.%20(2002))  Sweden  1982-1995 | NS | National board of health and welfare, Swedish medical birth register, | IVF (101/5579)  Singleton (45/3183)  Controls (46/2014) | Based on ICD-10  Intellectual Disability and cerebral palsy | Naturally conceived  (119/11.241)  + Singleton (115/10,955) + Twin (83/4,037) | Adjusted for sex, year of birth, and birth hospital | The most common neurological diagnosis was cerebral palsy, for which children born after IVF had an increased risk of 3·7 (2·0–6·6), and IVF singletons of 2·8 (1·3–5·8). Suspected developmental delay was increased four-fold (1·9–8·3) in children born after IVF. Twins born after IVF did not differ from control twins with respect to risk of neurological sequelae. Low–birthweight and premature infants were more likely to need habilitation than fullterm babies. Maternal age did not affect risk. |

**Supplemental Table 4**. Statistical Summary of Meta-Analysis

| **Outcome** | **Risk Ratio (95% CI),**  **p-value** | **Heterogeneity**  **(I^2^),**  **p-value** | **Egger**  **p-val** | **Habord p-val** | **Peter**  **p-val** | **Number of Studies’ Data** |  |
| --- | --- | --- | --- | --- | --- | --- | --- |
| **ART vs Non ART (Control)** | | | | | | |  |
| Cerebral Palsy | 1.82 [1.41, 2.34], 0.00 | 19%, 0.29 | 0.702 | 0.723 | 0.469 | 5 | |
| Intellectual Disability | 1.01 [0.85, 1.21], 0.89 | 49%, 0.10 | 0.754 | 0.872 | 0.963 | 5 | |
| Autism Spectrum Disorder | 0.98 [0.86, 1.11], 0.74 | 0%, 0.72 | 0.290 | 0.836 | 0.506 | 5 | |
| Behavioral Problem | 1.04 [0.89, 1.21], 0.63 | 11%,0.29 | NE | NE | NE | 2 | |
| **Summary** | **1.05 [0.97, 1.14], 0.20** | **54%, 0.005** | **0.501** | **0.962** | **0.991** | **17** | |
|  |  |  |  |  |  |  | |
| **ICSI vs IVF (Control)** | | | | | | | |
| Cerebral Palsy | 0.83 [0.49, 1.42], 0.50 | 0%, 0.72 | NE | NE | NE | 2 | |
| Intellectual Disability | 1.46 [1.03, 2.08], 0.03 | 46%, 0.16 | 0.487 | 0.786 | 0.311 | 3 | |
| Autism Spectrum Disorder | 1.49 [1.05, 2.11], 0.03 | 0%,0.39 | NE | NE | NE | 2 | |
| **Summary** | **1.33 [1.06, 1.66], 0.01** | **27%, 0.22** | **0.061** | **0.574** | **0.116** | **7** | |
|  |  |  |  |  |  |  | |
| **Frozen vs Fresh (Control)** | | | | | | | |
| Cerebral Palsy | 0.97 [0.55, 1.73], 0.93 | 13%, 0.32 | 0.974 | 0.601 | 0.186 | 3 | |
| Intellectual Disability | 0.88 [0.63, 1.22], 0.44 | 0%, 0.91 | 0.619 | 0.937 | 0.293 | 3 | |
| Autism Spectrum Disorder | 0.81 [0.63, 1.05], 0.12 | 0%, 0.76 | NE | NE | NE | 2 | |
| **Summary** | **0.85 [0.70, 1.03], 0.09** | **0%, 0.87** | **0.140** | **0.882** | **0.295** | **8** | |
|  |  |  |  |  |  |  | |
| **COFOUNDER EFFECTS** | | | | | | | |
| **Low Birthweight** |  |  |  |  |  |  | |
| Cerebral Palsy | 2.33 [2.18, 2.50], 0.00 | 96%, 0.00 | NE | NE | NE | 2 | |
| Intellectual Disability | 1.67 [1.50, 1.86], 0.00 | NE | NE | NE | NE | 2 | |
| Autism Spectrum Disorder | 1.59 [1.43, 1.77], 0.00 | 0%, 0.96 | 0.352 | 0.920 | 0.886 | 3 | |
| **Summary** | **1.80 [1.70, 1.91], 0.00** | **94%, 0.00** | **0.006** | **0.927** | **0.647** | **7** | |
|  |  |  |  |  |  |  | |
| **Preterm Birth** |  |  |  |  |  |  | |
| Cerebral Palsy | 3.01 [2.80, 3.24], 0.00 | 0%, 0.44 | NE | NE | NE | 2 | |
| Intellectual Disability | 2.55 [2.44, 2.66], 0.00 | 96%, 0.00 | NE | NE | NE | 1 | |
| Autism Spectrum Disorder | 1.61 [1.51, 1.71], 0.00 | 92%, 0.00 | NE | NE | NE | 2 | |
| **Summary** | **2.22 [2.15, 2.30], 0.00** | **98%, 0.00** | **0.005** | **0.926** | **0.646** | **5** | |
|  |  |  |  |  |  |  | |
| **ART vs Non-ART** |  |  |  |  |  |  | |
| Multiple birth | 14.57 [14.42, 14.73], 0.00 | 100%, 0.00 | 0.004 | 0.962 | 0.232 | 5 | |
| Preterm birth | 3.88 [3.82, 3.93], 0.00 | 99%, 0.00 | 0.004 | 0.953 | 0.967 | 5 | |
| Low birthweight | 5.66 [5.50, 5.82], 0.00 | 100%, 0.00 | 0.005 | 0.934 | 0.141 | 3 | |
| **Summary** | 6.93 [6.87, 6.98], 0.00 | **100%, 0.00** | **0.001** | **0.972** | **0.495** | **13** | |

**Supplemental Table 5**. Summary of findings and study quality assessment with GRADE approach of ART vs Non-ART studies

| **Participants**  **(No of studies)** | **QUALITY ASSESSMENT** | | | | | |  | **SUMMARY OF FINDINGS** | | | |
| --- | --- | --- | --- | --- | --- | --- | --- | --- | --- | --- | --- |
|  | Risk of bias | Inconsistency | Indirectness | Imprecision | Publication bias | Overall quality |  | ART | Non-ART | Relative effect (95% CI) | Absolute |
| **Neurodevelopmental Disorders** | | | | | | | | | | | |
| 8,027,256  (10 studies) | Not serious | Not serious | Not serious | Not serious | Undetected | ⊕⊕⊕⊖  MODERATE |  | 796/13,8161  (0.6%) | 43,013/7,889,095  (0.5%) | **1.05** (0.97 to 1.14) | **0 more per 1000**  (from 0 more to 1 more)  **Non-ART Risk**  **5 per 1000** |
| **Cerebral Palsy** | | | | | | | | | | | |
| 628,067  (5 studies) | Not serious | Not serious | Not serious | Not serious | Undetected | ⊕⊕⊕⊖  MODERATE |  | 97/22,139  (0.44%) | 2,325/605,928  (0.38%) | **1.82** (1.41 to 2.34) | **3 more per 1000**  (from 2 more to 5 more)  **Non-ART Risk**  **4 per 1000** |
| **Intellectual Disability** | | | | | | | | | | | |
| 3,139,382  (5 studies) | Not serious | Not serious | Not serious | Not serious | Undetected | ⊕⊕⊕⊖  MODERATE |  | 131/45,263  (0.3%) | 10,646/3,094,119  (0.3%) | **1.01** (0.85 to 1.21) | **0 more per 1000**  (from 1 fewer to 1 more)  **Non-ART Risk**  **3 per 1000** |
| **Autism Spectrum Disorder** | | | | | | | | | | | |
| 3,695,552  (6 studies) | Not serious | Not serious | Not serious | Not serious | Undetected | ⊕⊕⊕⊖  MODERATE |  | 406/59,075  (0.7%) | 20,250/3,636,477  (0.6%) | **0.98** (0.86 to 1.11) | **0 fewer per 1000**  (from 1 fewer to 1 more)  **Non-ART Risk**  **6 per 1000** |
| **Behavioral Problems** | | | | | | | | | | | |
| 564,225  (2 studies) | Not serious | Not serious | Not serious | Not serious | Undetected | ⊕⊕⊕⊖  MODERATE |  | 162/11,684  (1.4%) | 9,792/552,571  (1.8%) | **1.04** (0.89 to 1.21) | **1 more per 1000**  (from 2 more to 4 more)  **Non-ART Risk**  **18 per 1000** |

**Supplemental Table 6**. Summary of findings and study quality assessment with GRADE approach of ICSI vs IVF studies

| **Participants**  **(No of studies)** | **QUALITY ASSESSMENT** | | | | | |  | **SUMMARY OF FINDINGS** | | | |
| --- | --- | --- | --- | --- | --- | --- | --- | --- | --- | --- | --- |
|  | Risk of bias | Inconsistency | Indirectness | Imprecision | Publication bias | Overall quality |  | ICSI | IVF | Relative effect (95% CI) | Absolute |
| **Neurodevelopmental Disorders** | | | | | | | | | | | |
| 107,279  (5 studies) | Not serious | Not serious | Not serious | Not serious | Undetected | ⊕⊕⊕⊖  MODERATE |  | 181/50,176  (0.36%) | 165/57,103  (0.29%) | **1.38** (1.1 to 1.73) | **1 more per 1000**  (from 0 more to 2 more)  **IVF (control) Risk**  **3 per 1000** |
| **Cerebral Palsy** | | | | | | | | | | | |
| 19,499  (2 studies) | Not serious | Not serious | Not serious | Not serious | Undetected | ⊕⊕⊕⊖  MODERATE |  | 18/5,491  (0.33%) | 54/14,008  (0.39%) | **0.83** (0.49 to 1.42) | **1 fewer per 1000**  (from 2 fewer to 2 more)  **IVF (control) risk**  **4 per 1000** |
| **Intellectual Disability** | | | | | | | | | | | |
| 39,094  (2 studies) | Not serious | Not serious | Not serious | Not serious | Undetected | ⊕⊕⊕⊖  MODERATE |  | 56/13,571  (0.41 %) | 71/25523  (0.28%) | **1.46** (1.03 to 2.08) | **2 more per 1000**  (from 0 more to 5 more)  **IVF (control) Risk**  **3 per 1000** |
| **Autism Spectrum Disorder** | | | | | | | | | | | |
| 59,899  (2 studies) | Not serious | Not serious | Not serious | Not serious | Undetected | ⊕⊕⊕⊖  MODERATE |  | 110/34,763  (0.32%) | 52/25,136  (0.21%) | **1.49** (1.05 to 2.11) | **1 more per 1000**  (from 0 more to 2 more)  **IVF (control) Risk**  **2 per 1000** |

**Supplemental Table 7**. Summary of findings and study quality assessment with GRADE approach of Frozen vs Fresh Embryo Transfer studies

| **Participants**  **(No of studies)** | **QUALITY ASSESSMENT** | | | | | |  | **SUMMARY OF FINDINGS** | | | |
| --- | --- | --- | --- | --- | --- | --- | --- | --- | --- | --- | --- |
|  | Risk of bias | Inconsistency | Indirectness | Imprecision | Publication bias | Overall quality |  | Frozen | Fresh | Relative effect (95% CI) | Absolute |
| **Neurodevelopmental Disorders** | | | | | | | | | | | |
| 138,835  (6 studies) | Not serious | Not serious | Not serious | Not serious | Undetected | ⊕⊕⊕⊖  MODERATE |  | 135/18,764  (0.72%) | 1970/120,071  (1.6%) | **0.85** (0.7 to 1.03) | **2 fewer per 1000**  (from 5 fewer to 0 more)  **Fresh (control) Risk**  **16 per 1000** |
| **Cerebral Palsy** | | | | | | | | | | | |
| 20,796  (3 studies) | Not serious | Not serious | Not serious | Not serious | Undetected | ⊕⊕⊕⊖  MODERATE |  | 10/1,243  (0.8%) | 1,300/19,553  (6.6%) | **0.97** (0.55 to 1.73) | **2 fewer per 1000**  (from 30 fewer to 49 more)  **Fresh (control) risk**  **66 per 1000** |
| **Intellectual Disability** | | | | | | | | | | | |
| 44,880  (3 studies) | Not serious | Not serious | Not serious | Not serious | Undetected | ⊕⊕⊕⊖  MODERATE |  | 58/6,895  (0.84 %) | 197/37,985  (0.52%) | **0.88** (0.63 to 1.22) | **1 fewer per 1000**  (from 2 fewer to 1 more)  **Fresh (control) Risk**  **5 per 1000** |
| **Autism Spectrum Disorder** | | | | | | | | | | | |
| 73,159  (2 studies) | Not serious | Not serious | Not serious | Not serious | Undetected | ⊕⊕⊕⊖  MODERATE |  | 67/62,533  (0.76%) | 67/10,626  (0.63%) | **0.81** (0.63 to 1.05) | **1 fewer per 1000**  (from 3 fewer to 0 more)  **Fresh (control) Risk**  **8 per 1000** |

**Supplemental Table 8**. Summary of findings and study quality assessment with GRADE approach of Confounder Effects studies

| **Participants**  **(No of studies)** | **QUALITY ASSESSMENT** | | | | | |  | **SUMMARY OF FINDINGS** | | | |
| --- | --- | --- | --- | --- | --- | --- | --- | --- | --- | --- | --- |
|  | Risk of bias | Inconsistency | Indirectness | Imprecision | Publication bias | Overall quality |  | With Confounder Effect | Control | Relative effect (95% CI) | Absolute |
| **PRETERM BIRTH – Neurodevelopmental Disorders** | | | | | | | | | | | |
| 6,212,542  (7 studies) | Not serious | Not serious | Not serious | Not serious | Reporting bias strongly suspected^1^ | ⊕⊕⊖⊖  LOW^1,2^ |  | 4,116/356,354  (1.2%) | 27,547/5,856,188  (0.5%) | **2.22** (2.15 to 2.30) | **6 more per 1000**  (from 5 fewer to 0 more)  **Control Risk**  **5 per 1000** |
| **PRETERM BIRTH - Cerebral Palsy** | | | | | | | | | | | |
| 407,700  (2 studies) | Not serious | Not serious | Not serious | Not serious | Undetected^1^ | ⊕⊕⊕⊖  MODERATE^1,2^ |  | 517/15,230  (3.4%) | 1,657/392,470  (0.42%) | **3.01** (2.8 to 3.24) | **8 more per 1000**  (from 8 more to 9 more)  **Control risk**  **4 per 1000** |
| **PRETERM BIRTH - Intellectual Disability** | | | | | | | | | | | |
| 2,688,104  (2 studies) | Not serious | Not serious | Not serious | Not serious | Reporting bias strongly suspected^1^ | ⊕⊕⊕⊖  LOW^1,2^ |  | 2,396/138,289  (1.7%) | 16,424/2,549,815  (0.6%) | **2.55** (2.44 to 2.66) | **10 more per 1000**  (from 2 fewer to 1 more)  **Control Risk**  **6 per 1000** |
| **PRETERM BIRTH - Autism Spectrum Disorder** | | | | | | | | | | | |
| 3,116,738  (3 studies) | Not serious | Not serious | Not serious | Not serious | Reporting bias strongly suspected^1^ | ⊕⊖⊖⊖  VERY LOW^1,2^ |  | 1,203/202,835  (0.6%) | 9,466/2,913,903  (0.3%) | **1.61** (1,51 to 1.71) | **2 more per 1000**  (from 2 more to 2 more)  **Control**  **3 per 1000** |
|  |  |  |  |  |  |  |  |  |  |  |  |
|  |  |  |  |  |  |  |  |  |  |  |  |
| **LOW BIRTHWEIGHT/ SMALL FOR GESTATIONAL AGE – Neurodevelopmental Disorders** | | | | | | | | | | | |
| 1,234,512  (5 studies) | Not serious | Not serious | Not serious | Not serious | Reporting bias strongly suspected^1^ | ⊕⊖⊖⊖  VERY LOW^1,2^ |  | 1,138/66,226  (1.7%) | 8,960/1,168,286  (0.8%) | **1.8** (1.7 to 1.91) | **6 more per 1000**  (from 5 more to 7 more)  **Control Risk**  **8 per 1000** |
| **LOW BIRTH WEIGHT/SMALL FOR GESTATIONAL AGE - Cerebral Palsy** | | | | | | | | | | | |
| 406,450  (2 studies) | Not serious | Not serious | Not serious | Not serious | Undetected^1^ | ⊕⊕⊖⊖  LOW^1,2^ |  | 348/14,952  (2.3%) | 1,826/391,498  (0.47%) | **2.33** (2.18 to 2.5) | **6 more per 1000**  (from 6 more to 7 more)  **Control risk**  **5 per 1000** |
| **LOW BIRTH WEIGHT/ SMALL FOR GESTATIONAL AGE - Intellectual Disability** | | | | | | | | | | | |
| 209,904  (1 study) | Not serious | Not serious | Not serious | Not serious | Reporting bias strongly suspected^1^ | ⊕⊖⊖⊖  VERY LOW^1,2^ |  | 350/11708 (3%) | 3,551/198,196  (1.8%) | **1.67** (1.5 to 1.86) | **12 more per 1000**  (from 9 more to 15 more)  **Control Risk**  **18 per 1000** |
| **LOW BIRTH WEIGHT/SMALL FOR GESTATIONAL AGE - Autism Spectrum Disorder** | | | | | | | | | | | |
| 618,158  (2 studies) | Not serious | Not serious | Not serious | Not serious | Reporting bias strongly suspected^1^ | ⊕⊕⊖⊖  LOW^1,2^ |  | 440/39,566 (1.1%) | 3583/578,592 (0.62%) | **1.59** (1.43 to 1.77) | **4 more per 1000**  (from 3 more to 5 more)  **Control**  **6 per 1000** |
|  |  |  |  |  |  |  |  |  |  |  |  |

^1^ Risk of publication bias based on I^2^ heterogeneity test and Egger’s test

^2^ Other confounder effects, multiple birth, caused the increase risk. In some studies, singleton and multiple birth data cannot be separated for confounder effect analysis

**Supplemental Table 9**. Summary of findings and study quality assessment with GRADE approach of Risk of Multiple Birth, Preterm Birth, Low birth body weight in ART and Non-ART children

| **Participants**  **(No of studies)** | **QUALITY ASSESSMENT** | | | | | |  | **SUMMARY OF FINDINGS** | | | |
| --- | --- | --- | --- | --- | --- | --- | --- | --- | --- | --- | --- |
|  | Risk of bias | Inconsistency | Indirectness | Imprecision | Publication bias | Overall quality |  | Frozen | Fresh | Relative effect (95% CI) | Absolute |
| **Multiple Birth** | | | | | | | | | | | |
| 4,256,031  (5 studies) | Not serious | Not serious | Not serious | Not serious | Reporting bias strongly suspected^1^ | ⊕⊕⊕⊖  MODERATE^1^ |  | 26,224/62,045  (42.3%) | 119,266/4,193,986  (2.8%) | **14.57** (14.42 to 14.73) | **386 more per 1000**  (from 382 more to 390 more)  **Non-ART risk**  **28 per 1000** |
| **Preterm Birth** | | | | | | | | | | | |
| 4,192,333  (5 studies) | Not serious | Not serious | Not serious | Not serious | Reporting bias strongly suspected^1^ | ⊕⊕⊕⊖  MODERATE^1,2^ |  | 16,282/72,442 (22.5%) | 238,284/4,119,891  (5.8 %) | **3.88** (3.82 to 3.93) | **167 more per 1000**  (from 163 more to 169 more)  **Non-ART Risk**  **58 per 1000** |
| **Low birth body weight** | | | | | | | | | | | |
| 765,205  (3 studies) | Not serious | Not serious | Not serious | Not serious | Reporting bias strongly suspected^1^ | ⊕⊕⊕⊖  MODERATE^1,2^ |  | 5,242/25,376  (20.7 %) | 34,616/739,629  (4.7%) | **5.66** (5.5 to 5.82) | **219 more per 1000**  (from 212 more to 227 more)  **Non-ART Risk**  **47 per 1000** |

^1^ Risk of publication bias based on I^2^ heterogeneity test and Egger’s test

^2^ Other confounder effects, multiple birth, caused the increase risk. In some studies, singleton and multiple birth data cannot be separated for confounder effect analysis


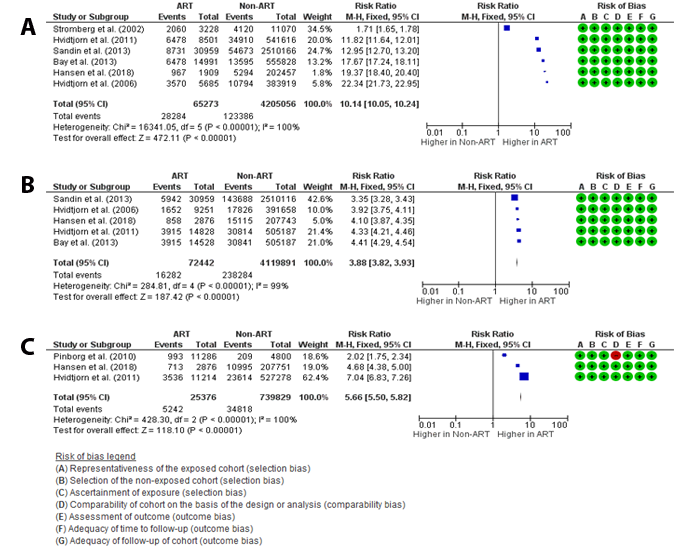


**Supplemental Figure 1** Forest plots show the results of meta-analysis on risk of multiple birth (**A**), preterm birth (**B**) and low birth body weight (**C**) in ART and non-ART children. Meta-analysis showed that the confounder factors are higher in ART.
